# Functional Roles of Non-coding RNAs in the Interaction Between Host and Influenza A Virus

**DOI:** 10.3389/fmicb.2021.742984

**Published:** 2021-10-22

**Authors:** Nelam Sajjad, Song Wang, Ping Liu, Ji-Long Chen, Xiaojuan Chi, Shasha Liu, Shujie Ma

**Affiliations:** ^1^Key Laboratory of Fujian-Taiwan Animal Pathogen Biology, College of Animal Sciences, Fujian Agriculture and Forestry University, Fuzhou, China; ^2^CAS Key Laboratory of Pathogenic Microbiology and Immunology, Institute of Microbiology, Chinese Academy of Sciences, Beijing, China

**Keywords:** non-coding RNAs, influenza A virus, infection, innate immunity, interferons

## Abstract

Non-coding RNAs (ncRNAs) are extensively expressed in various cells and tissues, and studies have shown that ncRNAs play significant roles in cell regulation. However, in the past few decades, the knowledge of ncRNAs has been increased dramatically due to their transcriptional ability and multiple regulatory functions. Typically, regulatory ncRNAs include long ncRNAs (lncRNAs), miRNAs, piRNAs, Y RNAs, vault RNAs, and circular RNAs (circRNAs), etc. Previous studies have revealed that various ncRNAs are involved in the host responses to virus infection and play critical roles in the regulation of host-virus interactions. In this review, we discuss the conceptual framework and biological regulations of ncRNAs to elucidate their functions in response to viral infection, especially influenza A virus (IAV) infection. In addition, we summarize the ncRNAs that are associated with innate immunity and involvement of interferons and their stimulated genes (ISGs) during IAV infection.

## Introduction

DNA, together with RNA, is the fundamental carrier of genetic information. Transcriptomic analysis has revealed that 70–90% genome is transcribed, in which only approximately 2% is translated into protein (Djebali et al., 2012). Researchers have long focused on mRNAs that bear protein-coding capacity, while non-coding RNAs (ncRNAs) have been widely shown as “Junk” transcriptional byproducts with less meanings. However, ncRNAs which are transcribed from DNA but not encode proteins carry crucial biological information. From the late 1950s, the era of the rRNAs and tRNAs discovery has gradually opened the knowledge world of ncRNAs, since then many studies show that ncRNAs participate in the regulation of gene expression at the levels of transcription, RNA processing, and translation (Cech and Steitz, 2014). Generally, ncRNAs are involved in regulating biological, developmental, and physiological processes, even serving as molecular markers of some diseases (Pavet et al., 2011; Salzman, 2016; Adams et al., 2017; Zhu et al., 2018; Liu C. X. et al., 2019). Besides, the expression pattern of ncRNAs usually changes in response to various viral infections, indicating that ncRNAs play important roles in modulating viral infection by different mechanisms (Li et al., 2015; Ouyang et al., 2016; Liu Z. et al., 2021; Othumpangat et al., 2021).

Influenza virus is mainly classified into four subtypes based on the antigenicities of nucleic protein (NP) and matrix protein (M), including influenza A virus (IAV), influenza B virus (IBV), influenza C virus (ICV), and influenza D virus (IDV) (Hause et al., 2013; Long et al., 2019). IAV belongs to the *Orthomyxoviridae* family and its genome consists of eight negative single-stranded RNAs. IAVs are important zoonotic pathogens that can infect diverse host species including birds, pigs, and humans, causing mild to severe respiratory disorders and posing continuous threats to public health. It is known as one of the dominant pathogens that has led annual economic losses through seasonal epidemics and causing four major epidemics (1918, 1957, 1968, and 2009) worldwide (König et al., 2010). In addition, IAVs are further classified into 18 hemagglutinin (HA) and 11 neuraminidase (NA) in animals and humans according to the genetic and antigenic variability of the two surface proteins, while H17N10 and H18N11 are only identified in bats (Tong et al., 2013; Wu et al., 2014). Typically, IAVs, entering the host through nasal or oral pathway, are usually countered by the mucus that covers the respiratory epithelium. Once the viruses successfully get through the mucous layer, the HA receptor-binding site immediately attaches the virus to cell surface receptors (α2,3- or α2,6-linked sialic acid residues) to facilitate the internalization of virus by endocytosis. In the process of endosomal trafficking, pH-dependent fusion of viral and endosomal membranes leads to release of viral ribonucleoproteins (vRNPs) into the cellular cytoplasm. Then, the released vRNPs are transported into nucleus to initiate replication and transcription of viral RNAs for production of progeny viruses. These events can trigger multiple machineries in the interaction between IAVs and hosts. Thus, the host factors including ncRNAs and proteins are usually required in the regulation of viral replication in the process of IAV infection.

Host antiviral immune responses are stimulated once the viruses invade host cells. Innate immunity comes as the first defense line against the viral infection (Gu et al., 2019). Upon infection of IAV, the host innate immunity is activated through recognition of pathogen-associated molecular patterns (PAMPs) by pattern recognition receptors (PRRs). Studies have shown that IAV can be recognized by multiple classes of PRRs of the innate immune system, including the Toll-like receptors (TLR3, TLR7, and TLR8), retinoic acid-inducible gene I (RIG-I), and the NOD-like receptor family member NOD-, LRR-, and pyrin domain-containing 3 (NLRP3). Various PAMPs generated from IAVs such as double-stranded RNA (dsRNA, sensed by TLR3), single-stranded RNA (ssRNA, sensed by TLR7 and TLR8), and 5′-triphosphate RNA (sensed by RIG-I) lead to the induction of the expression of nuclear factor-κB (NF-κB), IRF3, or IRF7 to induce a large number of cellular cytokines to fight with viruses (Rehwinkel et al., 2010; Biondo et al., 2019). In addition, the Matrix ion channel activity of IAV in the Golgi can promote the formation of NLRP3 inflammasome, resulting in the release of cytokines interleukin-1β (IL-1β) and IL-18 (Pang and Iwasaki, 2012). These cytokines, generated by the host, can bind with receptors to activate the JAK-STAT pathway, leading to the transcription of interferon-stimulated genes (ISGs), which exert a broad antiviral spectrum. Consequently, signaling cascades induced by viral proteins or viral nucleic acids prevent viral replication and establish antiviral status (Darnell et al., 1994; Li et al., 2019). Many studies have revealed that ncRNAs function as negative or positive regulators in the host antiviral immunity through various mechanisms in the process of viral infection (Pedersen et al., 2007; Umbach and Cullen, 2009; Vierbuchen and Fitzgerald, 2021). In this review, we summarize the current roles of ncRNAs, typically including long ncRNAs (lncRNAs), miRNAs, piRNAs, Y RNAs, vault RNAs (vtRNAs), and circular RNAs (circRNAs), especially in their functions as regulators in fighting with IAV infection through innate immunity pathways.

## Characteristics and Modes of Action of Non-Coding RNAs

Exciting outcomes reveal that discovery of higher eukaryotic transcriptome has opened a new door for the study of ncRNAs (Rinn and Chang, 2012; Morris and Mattick, 2014). In eukaryotes, various ncRNAs are produced during transcription and RNA processing in different genomic regions of eukaryotes. Specific regions of DNAs could be transcribed into ncRNAs, including transposon elements, protein coding genes, and enhancer regions. Regulatory ncRNAs (lncRNAs, miRNAs, piRNAs, Y RNAs, vtRNAs, and circRNAs, etc.) are expressed at certain stages (transcriptional, post-transcriptional, or epigenetic levels) and can modulate the expression of other genes at the level of transcription or translation.

LncRNAs are designated as transcripts bearing over 200 nt in length with no protein-coding capacity. Based on their locations in the genome, lncRNAs can be divided into sense, antisense, bidirectional, intronic, and intergenic lncRNAs (Rinn and Chang, 2012; St Laurent et al., 2015). The currently annotated lncRNAs released from GENCODE in humans (version 35) and mice (version 25) are 17,957 and 13,197, respectively (Frankish et al., 2021). The number of annotated lncRNAs is continuing to increase, indicating the research hotspots and the critical roles of lncRNAs in the biological processes (Frankish et al., 2021). However, some of the interpreted lncRNAs contain open reading frame (ORF), which encode micropeptides, indicating that the accurate annotation and classification of lncRNAs need further to be studied (Pueyo et al., 2016; Bhatta et al., 2020). LncRNAs are involved in various biological processes such as transcriptional and post-transcriptional regulation, mRNA splicing, processing, transport, and translation (Sun et al., 2018). Further studies show that lncRNAs are involved in various cellular processes, including cell differentiation (Wang et al., 2014), cell growth (Wang et al., 2008), nuclear organization (Sun et al., 2018), cancer cell biology (Schmitt and Chang, 2016; Tang et al., 2018; Vafadar et al., 2019), and inactivation of X-chromosomes (Wutz, 2011). Additionally, lncRNAs can also act as competitive endogenous RNAs (ceRNAs) or miRNA sponges in the process of cancer (Poliseno et al., 2010; Shan et al., 2018; Kong et al., 2020) and virus infection (Chai et al., 2018) to protect target mRNAs from degradation. More importantly, various studies have confirmed that lncRNAs are involved in modulating immune responses against IAV infection (Table 1).

**TABLE 1:** Regulatory ncRNAs.

**ncRNAs**	**Influenza virus strains**	**Functions**	**Mechanisms**	**References**
LncRNA-VIN	A/WSN/1933 (H1N1)	Promote IAV infection	Affect viral protein synthesis.	Winterling et al., 2014
IPAN	A/WSN/1933 (H1N1) A/PR/8/34 (H1N1) A/Beijing/30/95 (H3N2)	Promote IAV infection	Form the IPAN/PB1 complex to prevent PB1 degradation.	Wang J. et al., 2019
LncRNA-PAAN	A/WSN/1933 (H1N1)	Promote IAV infection	Promotes the assemblage of RdRp complex and thus enhances polymerase activity of viral RNA.	Wang et al., 2018
TSPOAP1-AS1	A/Puerto Rico/8/1934 (H1N1)	Promote IAV infection	Represse IAV activated type I IFN signaling through negative induction of various key ISGs (IFIT2, IFIT3, IFITM3, OASL, and ISG20).	Wang Q. et al., 2019
Lnc-MxA	A/WSN/1933 (H1N1)	Promote IAV infection	An ISG which inhibits the transcription activation of IFN-β by forming triplex of DNA-RNA at promoter site.	Li et al., 2019
lncRNA ISR	A/WSN/1933 (H1N1)	Inhibit IAV infection	Enhance IFN-β production via RIG-I-dependent signaling pathway	Pan et al., 2019
NRAV	A/WSN/1933 (H1N1)	Promote IAV infection	Modulate negative antiviral immune response by suppressing initial transcription of various key ISGs (IFIT2, IFIT3, IFITM3, and OASL).	Ouyang et al., 2014
Lnc-ISG20	A/WSN/1933 (H1N1), A/Puerto Rico/8/1934 (H1N1), A/California/04/2009 (H1N1)	Inhibit IAV infection	Serves as a new ISG and binds with miR-326 which lessens the arbitration of miR-326 against inhibition of ISG20 expression.	Chai et al., 2018
LncRNA-155	A/WSN/1933 (H1N1) A/Puerto Rico/8/1934 (H1N1)	Inhibit IAV infection	Boost the innate immunity through PTP1B inhibition which involves to upregulate the several ISGs and IFN-β expression.	Maarouf et al., 2019
IVRPIE	A/Beijing/501/2009 (H1N1)	Inhibit IAV infection	Accelerate host antiviral immunity through positive regulation of ISGs and IFN-β expression by affecting histone modification of these genes.	Zhao et al., 2020
miR-223	A/PR/8/34 (H1N1) A/WSN/1933 (H1N1) A/OK/3052/09 (H3N2) A/OK/309/06 (H3N2)	Inhibit IAV infection	STIM1/miR-223/NLRP3 axis.	Liu C. C. et al., 2021
miR-21-3p	A/Hong Kong/156/97 (H5N1)	Promote H5N1 replication	Reduce FGF2 expression and confine type I IFN response to facilitate H5N1 replication.	Shi et al., 2020
miR-30	A/duck/Hubei/hangmei01/2006 (H5N1) A/Puerto Rico/8/34 (H1N1) A/duck/Hubei/W1/2004 (H9N2)	Inhibited IAV proliferation	Reduce SOCS1, SOCS3, and NEDD4 expression, and thus relieved their inhibiting effects on IFN/JAK/STAT and IFITM3 signaling pathway.	Lin et al., 2020
miR-93	A/PR/8/34 (H1N1)	Inhibit IAV infection	Downregulation of miR-93 strengthens IFN-JAK-STAT pathway via JAK1 upregulation to inhibit IAV infection.	Guo et al., 2020
miR-29a	A/Puerto Rico/8/34 (H1N1) A/Oklahoma/3052/09 (H1N1) A/WSN/1933 (H1N1) A/Oklahoma/309/2006 (H3N2)	Inhibit IAV infection	Inhibit virus replication through targeting the Wnt-Ca2 + signaling receptor frizzled 5 protein.	Yang et al., 2021
miR-206	A/Puerto Rico/8/34 (H1N1) A/WSN/1933 (H1N1), A/Oklahoma/3052/2009 (H1N1) A/Oklahoma/309/2006 (H3N2)	Inhibit IAV infection	Target TNKS2 and activates JNK/c-Jun signaling, inducing type I interferon expression and enhanced STAT signaling.	Bamunuarachchi et al., 2021
miR-101	A reassortant between PR8 and A/Aichi/68(H3N2)	Inhibit IAV infection	Upregulation of miR-101 restrains the mTOR expression to inhibit IAV propagation.	Sharma et al., 2020
miR-483-3p	A/Puerto Rico/8/34 (H1N1) Mouse adapted-A/California/04/09 (H1N1) A/Anhui/1/13 (H7N9) A/Vietnam/1203/04 (H5N1)	Inhibit IAV infection	Promote innate immune responses to IAV infection by targeting negative regulators of the RIG-I signaling pathway.	Maemura et al., 2018
miRNA-192	Rescued virus of HA from H5 and backbone from A/PR/8/34 (H1N1)	Attenuate the pathogenicity of IAV	Attenuate the pathogenicity by inserting miRNA into genome of IAV	Langlois et al., 2013
miRNA-192-5p	A/PR/8/34 (H1N1)	Attenuate the pathogenicity of IAV	Attenuate the pathogenicity by inserting miRNA into genome of IAV.	Gao et al., 2020
ssc-miR-221-3p	A/duck/Anhui/1/2006 (H5N1) A/chicken/Shandong/l × 1023/2007 (H9N2) 406 A/chicken/Hebei/F1027/2017 (H7N9) A/swine/Shandong/436/2012 (H1N1) A/Swine/Guangdong/201/2006 (H3N2)	Inhibit IAV infection	Host barrier during cross-species infection through NF-κB and its phosphorylation at position 65.	Song et al., 2020
ssc-miR-222	A/duck/Anhui/1/2006 (H5N1) A/chicken/Shandong/l × 1023/2007 (H9N2) 406 A/chicken/Hebei/F1027/2017 (H7N9) A/swine/Shandong/436/2012 (H1N1) A/Swine/Guangdong/201/2006 (H3N2)	Inhibit IAV infection	Host barrier during cross-species infection through NF-κB and its phosphorylation at position 65.	Song et al., 2020
vtRNA	A/PR/8/34 (H1N1) A/WSN/1933 (H1N1)	Promote IAV infection	Promoted viral replication by attenuating protein kinase R (PKR) activity.	Li et al., 2015
circ-GATAD2A	A/Puerto Rico/8/34 (H1N1)	Promote IAV infection	Accelerate IAV replication by inhibiting autophagy.	Yu et al., 2019
circ_0050463	A/PR/8/34 (H1N1) A/WSN/33(H1N1) A/Lufang/9/93 (H3N2)	Promote IAV infection	Function as an endogenous microRNA-33b-5p sponge to inhibit miR-33b-5p activity, resulting in increased eukaryotic translation elongation factor 1 alpha 1 (EEF1A1) expression.	Shi et al., 2021
AIVR	A/chicken/Jiangsu/C4258/2012 (H9N2) A/Anhui/1/2005 (H5N1) A/Anhui/1/2013 (H7N9)	Inhibit IAV infection	Absorb the miR-330-3p that binds the mRNA of CREBBP to work as a miRNA sponge, leading to large expression of CREBBP and accelerating IFN-β production.	Qu et al., 2021

MiRNAs, generated from hairpin loop-like transcripts, are the most abundant and well-studied class of short ncRNAs (Reinhart et al., 2000; Lagos-Quintana et al., 2001). MiRNAs have profound effects against viral immunity by affecting mRNA inhibition and silencing of target genes (Pedersen et al., 2007; Umbach and Cullen, 2009; Table 1). Most mammalian miRNAs are transcribed by RNA polymerase II as primary RNA (pri-miRNA), which is cleaved into precursor miRNA (pre-miRNA) by RNase III Drosha to form a stem-loop in the nucleus (Bartel, 2004). Then, the pre-miRNAs are transported to cytosol by exportin-5 and are cleaved by Dicer into miRNA duplex. One arm of the miRNA duplex binds with Argonaute (AGO) protein in the RNA-induced silencing complex (RISC) to act as a guide sequence for targeted mRNAs to silence genes by inducing mRNA degradation or inhibiting the translation (Krol et al., 2010).

However, unlike miRNAs, piRNAs bind to PIWI protein (a specific member of the AGO family) to form a piRNAs-induced silencing complex, resulting in silencing the transcriptional and post-transcriptional target points (Siomi et al., 2011). Y-RNAs are another regulatory RNAs involved in cellular stress response, RNA stability, and DNA replication (Christov et al., 2006; Kowalski and Krude, 2015). Recent studies have revealed that Y-RNAs performed critical roles in host defense against viral infection through exosome delivery (Liu Y. M. et al., 2019).

VtRNAs are barrel-shaped ncRNAs, commonly found in eukaryotic cells, which contain large numbers of ribonucleoprotein particles called vaults (Kedersha and Rome, 1986; Kedersha et al., 1991). Most vtRNAs exist in a free form and function in a non-structural way (Kickhoefer et al., 1998; Nandy et al., 2009), which are involved in the interaction between virus and host, especially by regulating the innate immune response (Li et al., 2015; Table 1).

Circular RNAs (circRNAs), firstly identified in Sendai virus and plant viroids, form covalently closed rings structurally and are produced by pre-mRNA back-splicing during post-transcriptional processing. Advanced deep sequence analysis drafted uniqueness of the circRNAs and found that circRNAs ranged in size from 100 to 10,000 nt (Salzman et al., 2012; Jeck et al., 2013; Ye et al., 2015). In terms of their unique ring structure, circRNAs have no terminated 5′ end caps or 3′ poly (A) tails, thus indicating the highly stable nuclease resistance (Starke et al., 2015). Accumulating evidences have indicated that circRNAs play vital roles in the regulation of IAV infection (Yu et al., 2019; Liu Z. et al., 2021; Shi et al., 2021; Table 1).

## Functional Involvement of Long Non-Coding RNAs in Regulation of Influenza a Virus Infection

Recent studies on lncRNAs have reported their interactions with immune responses to various diseases. Some lncRNAs have also been studied under the influence of IAV, but the exact nature of these lncRNAs remains unclear. It is known that thousands of different lncRNAs were expressed to control IAV infection, some of which directly or indirectly affect the functional roles of viral protein or alter cell metabolism by regulating the immune system. From the mechanical perspective, this study emphasizes the role of specific lncRNAs in the pathogenesis of IAVs.

## Differential Expression of Long Non-Coding RNAs During Influenza a Virus Infection

The IAV manipulates host factors to reduce the antiviral response and promote viral replication, resulting in a large number of infections. The analysis of differential expression of lncRNAs in IAV infection showed that most lncRNAs had similar regulatory effects in IAV, but differentially expressed in SARS-CoV infection (Peng et al., 2010). These studies showed that the differential expression of lncRNAs in the process of viral infection was controlled by innate immunity signaling (Peng et al., 2010). Additionally, a similar discovery was made by Josset et al. (2014) who performed RNA sequence on 8 different strains of mice and found 5,329 differentially expressed lncRNAs after IAV infection. Furthermore, sequence analysis of human lung cells (A549) was used to investigate different response pathways during IAV infection, and the expression of lncRNAs under different conditions was also reported. These lncRNAs have been studied by regulating autophagy, cellular metabolic reactions, and immune response, and are involved in IFNs signaling pathways of IAV infection (Ouyang et al., 2016; More et al., 2019; Unfried and Fortes, 2020). Taken together, these studies established a strong association between IAV infection and lncRNAs expression, suggesting that the expression patterns of lncRNAs after infection of IAV might be an effective diagnostic tool in clinical trials. During viral infection, lncRNAs act as regulators and participate in regulation, thereby inhibiting or promoting viral replication.

## Regulatory Roles of Long Non-Coding RNAs in Influenza a Virus Infection

LncRNA transcripts are larger than 200 nt and are involved in the complexity of genome evolution (Amaral et al., 2008; Ponting et al., 2009). Several studies unraveled the functional mechanisms of regulatory lncRNAs, including LncRNA-VIN (Winterling et al., 2014), IPAN (Wang J. et al., 2019), LncRNA-PAAN (Wang et al., 2018), PSMB8-AS1 (More et al., 2019), TSPOAP1-AS1 (Wang Q. et al., 2019), lncRNA ISR (Pan et al., 2019), Lnc-MxA (Li et al., 2019), LncRNA-155 (Maarouf et al., 2019), NRAV (Ouyang et al., 2014), IVRPIE (Zhao et al., 2020), and Lnc-ISG20 (Chai et al., 2018; Table 1). These lncRNAs have negative or positive roles in the regulation of viral replication by exerting multiple mechanisms.

LncRNAs can directly modulate IAV replication through targeting viral proteins. LncRNA-VIN is upregulated in the process of IAV infection. The silencing of lncRNA-VIN results in the reduction of viral protein synthesis of HA, NP, NS1, and M2, thereby inhibiting the production of IAV (Winterling et al., 2014). LncRNAs can also directly target viral proteins to regulate IAV replication. Loss-of-function screening analysis reveals that IPAN, an influenza virus PB1-associated ncRNA, is induced by IAV infection and promotes IAV infection by preventing PB1 degradation (Wang J. et al., 2019). Knock down the expression of lncRNA-PAAN can significantly impair the polymerase activity of IAV, resulting in the reduction of progeny viruses. Further study shows that lncRNA-PAAN directly target viral PA protein of IAV to promote virus replication (Wang et al., 2018). PSMB8-AS1 can be induced by both IAV infection and IFN-β1. The reduction of this lncRNA leads not only to the decreased of mRNA expression of viral NP and NS1 genes but also to the decreased protein expression of NP, NS1, and PB1, resulting in the reduction of the progeny viruses (More et al., 2019). The proliferation of virus in host cells is a complicated dynamic process. It is well known that IAVs hijack cellular protein synthesis machinery to promote viral infection. However, the balance of infection between IAV and host might be tipped by lncRNAs through targeting different viral proteins. Considering that the large numbers of host lncRNAs, the detailed and specific mechanisms in viral regulation still need to be further studied.

**FIGURE 1:**
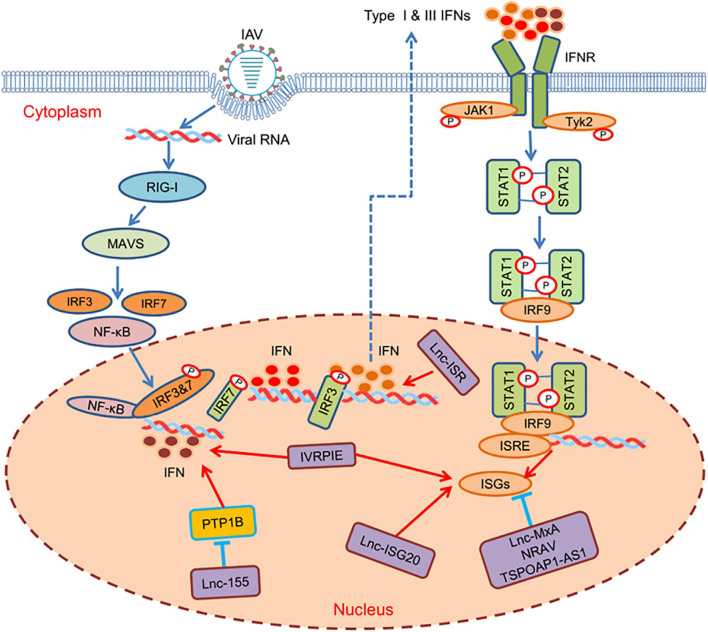
Host lncRNAs regulate various steps of antiviral immune responses against IAV infection. Upon IAV invading host cells, the pattern recognition receptors (PRRs) signaling pathway is initiated to activate transcription factors, including NF-κB and IRF3/7. Then, the transcription factors bind to the corresponding active sites to induce the transcription of IFNs, which activates auto- or paracrine pathways to initiate ISGs to defend viral infection through JAK-STAT pathways. Many lncRNAs are involved in the cascades to regulate different steps of the signaling pathway to inhibit or promote IAV replication.

In addition to directly target viral proteins, lncRNAs can function in antiviral immunity via regulating IFNs or ISGs. TSPOAP1-AS1 can be induced by IAV infection and IFN stimulus. It promotes IAV replication by decreasing IFN-β transcription, lowering ISGs expression, and suppressing the activation of interferon sensitive response elements (ISRE), however, the specific regulatory mechanism still needs to be determined (Wang Q. et al., 2019; Figure 1). LncRNA ISR, known as IFN-stimulated lncRNA, is significantly upregulated in the presence of IAV infection. During IAV infection, it enhances IFN-β production via RIG-I-dependent signaling pathway, thereby inhibiting viral replication (Pan et al., 2019; Figure 1). Lnc-MxA is a negative regulator of antiviral response and plays potent roles in immune homeostasis. It inhibits IFN-β transcription by the formation of a DNA-RNA complex at the IFN-β promoter site, thereby promoting IAV replication (Djebali et al., 2012; Figure 1). LncRNA-155, derived from MIR155HG, strongly increases the virulence and replication of IAV by indirectly affecting the expression of ISGs. In the process of IAV infection, protein tyrosine phosphatase 1B (PTP1B) is significantly inhibited by lncRNA-155, resulted in the increased expression of IFN-β and some key ISGs, thus guiding the immune response against IAV infection (Maarouf et al., 2019; Figure 1). NRAV, a downregulated lncRNA, can promote IAV replication and virulence *in vitro* and *in vivo*. Further study shows that NRAV can suppress the initial transcription of multiple ISGs such an IFITM3 and MxA through affecting histone modification of these genes (Ouyang et al., 2014; Figure 1). Recently, a novel lncRNA, IVRPIE, has been identified by transcriptome analysis in patients infected with IAV, which inhibits viral replication by promoting the transcription of ISGs (IFIT1, IFIT3, IRF1, Mx1, ISG15) and IFN-β. Furthermore, IVRPIE is associated with hnPNPU, which mediates the ISGs and IFN-β regulation through histone modification (Zhao et al., 2020; Figure 1). Similarly, lnc-ISG20, IAV-induced upregulated lncRNA, has a common sequence with ISG20 mRNA, which serves as a new ISG against IAV. In addition, it works as ceRNAs, binding to miR-326, promoting ISG20 mRNA transcription to ISG20 protein, and inhibiting viral replication (Chai et al., 2018; Figure 1). The influence of these lncRNAs on the negative or positive regulation of critical factors of innate immunity, including IFNs and ISGs, indicate that lncRNAs are crucial regulators in the process of IAV infection. Although large numbers of lncRNAs have been identified by using deep sequence, only a few of them have been characterized and studied during IAV infection. Considering that various host factors are involved in innate immunity, it is reasonable to speculate that there must exist more lncRNAs interacting with other transcriptional factors.

## Roles of Short Non-Coding RNAs in Influenza a Virus Infection

Short ncRNAs such as miRNAs, Y-RNAs, and vtRNAs utilize various molecular mechanisms in the regulation of IAV infection. Although the specific functional roles of miRNAs between host and virus interaction seem to be mysterious, the molecular mechanism of miRNA is clear, by hybridizing to target mRNAs and regulate their expression levels post-transcriptionally.

Typically, various studies have shown that miRNAs modulate the interaction of host and IAV through targeting genes of IAV or regulating host innate immune responses (Zhang et al., 2014; Chen X. et al., 2017; Guo et al., 2020; Lin et al., 2020; Shi et al., 2020; Liu C. C. et al., 2021). On the one hand, some miRNAs, such as hsa-miR-593-5p, hsa-miR-487b-5p, hsa-miR-486-5p, and hsa-miR-127-3p, were found to directly target gene segments of IAV and efficiently inhibited IAV replication (Peng et al., 2018). Similarly, miRNA-192 and miRNA-192-5p have shown the potential gene silencing ability to design novel live attenuated influenza vaccines by inserting the miRNA target sites into IAV genomes (Langlois et al., 2013; Gao et al., 2020). These studies show that miRNAs have potential to be used as diagnostic tool in IAV infection and promote the design of live attenuated IAV vaccines. On the other hand, multiple miRNAs are involved in the regulation of innate immunity during IAV infection. For example, IFN pathways usually are the primary targets in the regulation of miRNAs. The reduction of miR-21-3p in the patients’ serum and A549 cells infected with H5N1 resulted in the downregulation of FGF2, which facilitated the replication of H5N1 virus by impeding type I IFN response (Shi et al., 2020). In another study, miR-93 was downregulated in IAV infected alveolar epithelial cells, resulting in the promotion of IFN-JAK-STAT pathway via JAK1 upregulation, and enhanced the antiviral effect of type I IFN to suppress IAV infection (Guo et al., 2020). Liu C. C. et al. (2021) found that silencing STIM1 could promote the expression of miR-223, resulting in the alleviation of IAV-induced inflammation injury by inactivating NLRP3 and inflammasome, indicating that miR-223 was an important regulatory component in the STIM1/miR-223/NLRP3 axis. Lin et al. (2020) showed that miR-30 could inhibit the expression of SOCS1, SOCS3, and NEDD4, and thus positively enhanced type I IFN signaling pathway and IFITM3 expression, resulted in the reduction of progeny viruses of IAV. In addition, miRNAs can also influence IAV infection by regulating some host proteins, such as the Wnt-Ca^2+^ signaling receptor frizzled 5 (Yang et al., 2021), TNKS2 (Bamunuarachchi et al., 2021), and mTOR (Sharma et al., 2020). MiRNAs are also detected in exosomes, and they are involved in the host defense against IAV infection (Maemura et al., 2018; Hong et al., 2021; Othumpangat et al., 2021), indicating that these miRNAs might be the potential biomarkers for disease resistance. Previous studies have shown that both viral and host proteins participate in the regulation of host restriction of IAVs (Long et al., 2016; Liang et al., 2019; Zhang et al., 2020). However, in a recent study, Song et al. (2020) found that ssc-miR-221-3p and ssc-miR-222 were related to the host barrier during IAV interspecies infection, indicating that miRNAs played crucial roles in the host range restriction. Collectively, miRNAs are involved in the regulation of IAV infection through various molecular mechanisms including regulating IAV genes, host gens, or factors of the innate immunity. However, the interactions of IAVs and host genes or proteins are dynamic and balanced processes, and the regulatory patterns of miRNAs still need to be clarified in the future.

Y RNA-derived small RNAs (YsRNAs) are small fragments produced by degradation of Y RNAs. Although the nature and functions of YsRNAs remains unclear, studies show that YsRNA might be further processed into miRNA-like small RNAs, and might become potential biomarkers for breast cancer diagnosis (Dhahbi et al., 2014). In fact, hsa-miR-1975 has been proved to be derived from the degradation of Y5RNA in the process of apoptosis and shows antiviral effect against IAV infection (Liu Y. M. et al., 2019). However, the functional roles and molecular mechanisms of Y RNAs and YsRNAs during virus infection are still unknown. VtRNAs, together with vault proteins, form a ribonucleoprotein complex called vault particles. VtRNAs, including vtRNA1-1, vtRNA1-2, vtRNA1-3, and vtRNA2-1, are important components of vault particles (Kickhoefer et al., 1998). A recent study has shown that vault proteins regulate virus induced proinflammatory responses (Peng et al., 2016). Thus, it is not surprising that vtRNAs are differentially expressed after Epstein-Barr virus infection and may function in innate immune response to regulate virus replication (Mrázek et al., 2007; Nandy et al., 2009). Li et al. (2015) established a novel axis to clarify the mechanism of vtRNAs during IAV infection. Typically, IAVs evade the protein kinase R (PKR)-mediated innate immune antiviral response by upregulating vtRNAs through viral protein NS1, indicating that vtRNAs, as long as other ncRNAs, are also involved in the host-virus interaction through innate immunity (Li et al., 2015). However, considering the complex formed by vtRNAs and vault proteins, whether vault proteins or the whole complex can regulate IAVs or other viruses still need further study. Take together, these preliminary findings have shown that short ncRNAs as long as lncRNAs, play vital roles in the regulation of IAV infections. More importantly, some particular miRNAs might be helpful as a diagnostic or a therapeutic tool in the surveillance of IAVs. However, more solid and profound evidences are still required in the future.

## Roles of Circular RNAs in Virus Infection

Like other regulatory ncRNAs, circRNAs are initially misinterpreted as byproducts of RNA splicing that are produced form introns, exons, untranslated regions, intergenic regions, or tRNAs (Noto et al., 2017). However, recent studies show that circRNAs play important roles in the processes of biological regulation, including transcription and RNA splicing (Salzman, 2016), as well as function as miRNA sponges (Sun et al., 2016), and ceRNAs (Huang et al., 2016). Numerous circRNAs expressed genes are associated with enzyme activity, metal ion binding, nucleotide binding, and protein ubiquitination during reovirus infection (He et al., 2017). Furthermore, circRNAs are reported as disease diagnostic and predictive biomarkers (Qu et al., 2015) and they are also involved in the regulation of host-virus interaction (Awan et al., 2021).

The exogenous circRNAs can trigger immune response to protect the host from viral infection. Chen Y. G. et al. (2017) found that cells transfected with exogenous circRNAs had a 10-fold lower Venezuelan equine encephalitis virus-GFP (VEEV-GFP) infection rate. Furthermore, RIG-I co-aggregated with foreign circRNAs, which was necessary for detecting foreign circRNAs (Chen Y. G. et al., 2017). In another study, Li et al. (2017) demonstrated that circRNAs were involved in viral infection via dsRNA-binding proteins namely NF90 and NF110. Upon Poly(I:C) stimulation or Vesicular stomatitis virus (VSV) infection, circRNAs expression was decreased as a result of the export of NF90/NF110 to the cytoplasm. Meanwhile, NF90/NF110 was released from circRNA-protein complexes (circRNPs) and bound to viral mRNAs as part of their functions in antiviral immune response. The authors concluded a coordinated regulation of circRNAs biogenesis and function by NF90/NF110 in viral infection (Li et al., 2017). In a subsequent study by the same group, Liu C. X. et al. (2019) reported that circRNAs tended to form 16–26 bp imperfect RNA duplexes and act as inhibitors of PKR related to innate immunity. Upon poly(I:C) treatment or encephalomyocarditis virus (EMCV) infection, circRNAs are globally degraded, which is required for PKR activation. Meanwhile, circRNAs reduction and aberrant PKR activation are found in autoimmune disease systemic lupus erythematosus (SLE) (Liu C. X. et al., 2019). Yu et al. (2019) studied the role of circular RNA GATA Zinc Finger Domain Containing 2A (circ-GATAD2A) in the replication of IAV H1N1 in A549 cells. The authors detected high expression of circ-GATAD2A in response to IAV H1N1 infection. Knockdown of circ-GATAD2A in A549 cells enhanced autophagy and inhibited H1N1 replication. The authors concluded that circ-GATAD2A promoted the IAV replication by inhibiting autophagy (Yu et al., 2019; Table 1). Although numerous studies have characterized the expression profiling of circRNAs during virus infection and large numbers of circRNAs have been identified involving in the regulation of host-virus interactions (Liu Z. et al., 2021; Wang et al., 2021), the detailed molecular mechanisms are still limited and needs further investigation.

CircRNAs can modulate IAV infection by functioning as endogenous sponges to compete with miRNA for binding to mRNAs. For instance, circ_0050463 is up-regulated in IAV-infected A549 cells and knockdown of circ_0050463 in A549 cells inhibits IAV replication. Moreover, circ_0050463 acts as an endogenous sponge of microRNA-33b-5p to sequester and inhibits miR-33b-5p activity. Taken together, this study indicated that circRNA_0050463 facilitated IAV replication via miR-33b-5p/EEF1A1 axis (Shi et al., 2021; Table 1). AIVR, another novel intronic circRNA, mainly localizes in the cytoplasm and absorbs the miR-330-3p that binds the mRNA of CREBBP to work as a miRNA sponge, leading to large expression of CREBBP and accelerating IFN-β production (Qu et al., 2021; Table 1). Liu C. C. et al. (2021) reported that 7,126 circRNAs were expressed in the lungs of mice infected with H7N9 or H7N9 PB2-mutant viruses, of which 186 were differentially expressed. This result shows that differentially expressed circRNAs have vital roles in immune regulation against viral infection (Liu Z. et al., 2021).

Viral circRNAs have been identified in various classes of viruses including coronaviruses such as MERS-CoV, SARS-CoV-1, and SARS-CoV-2 (Yang et al., 2020; Cai et al., 2021), oncogenic viruses such as Epstein-Barr virus (EBV), Kaposi’s sarcomaassociated herpesvirus (KSHV), human papillomavirus (HPV), etc. (Avilala et al., 2021; Tagawa et al., 2021), as well as IAVs. However, to date, only a few of these viral circRNAs are functional identified. Some of these viral circRNAs act as miRNA sponges to regulate viral pathogenesis. In addition, viral circRNAs, detected in IAV infected cell lines or lungs, are available in Virus Circ Base, which will be helpful for the future study (Cai et al., 2020; Wang et al., 2021). Collectively, the discoveries of viral circRNAs are dramatically increased and gained more attention on these ncRNAs, which will be initiating an exciting new era of viral circRNAs.

## Conclusion and Perspectives

Although progresses have been made in understanding of the critical regulatory roles of ncRNAs against IAV infection, research in this field is still in its infancy stage. Some of the small ncRNAs, such as miRNAs and piRNAs modulate the virus-host interaction through silencing genes. However, most of other ncRNAs, including lncRNAs, circRNAs, and those small ncRNAs, take advantage of various molecular mechanisms to regulate the interactions between virus and host. The uncertainty of various mechanisms employed by ncRNAs makes it difficult for further research. Benefited from the deep sequencing technology and rapid advance of bioinformatics, sufficient evidence indicates that ncRNAs are crucial regulators in the complicated interaction network between the virus and host.

The infection caused by IAV poses continuous threats to human health and animal husbandry. Thus, the development of novel targets for therapeutics or biomarkers of IAV infection to promote the early diagnosis or treatment seems to be very important. Although accumulating evidences have demonstrated that ncRNAs are participated in the regulation of innate immune response, especially in the PRRs sensing pathway, the activation of transcription factors, the production of ISGs, and cell apoptosis, the possible regulation mechanisms of ncRNAs during IAV infection still need to be further studied. The intensive study of these ncRNAs will provide optional approaches for the prevention and control of IAVs. For instance, the expression of miRNAs in serum isolated from human patients with IAV infection shows different profiles, indicating the potential of miRNA as biomarkers. But for lncRNAs and circRNAs, the potential roles on the diagnosis need to be further studied with the limited molecular mechanism known. The altered expression or activity of these pivotal innate immune molecules significantly influences the host antiviral response and thereby affects the viral infection and replication. Interestingly, some ncRNAs acting as negative regulators of innate immunity can be hijacked by virus to inhibit the antiviral response, and some others functioning as positive regulators can be suppressed by virus during the infection. These findings provide strong evidences supporting the key role played by the ubiquitous and versatile lncRNAs in antiviral innate immunity. However, although thousands of ncRNAs are associated with viral infection, the number of ncRNAs with experimentally functional verified is limited. Therefore, intensive studies are still needed to define the expression, regulation and functioning of ncRNAs during virus infection. Overall, future studies tend to provide a more comprehensive picture of the interaction of host ncRNAs and viral infections, and shed light on the roles of ncRNAs in the antiviral response process.

## Author Contributions

NS, SL, and SM performed systematic literature review and wrote the manuscript. SW, PL, and XC revised the manuscript. J-LC organized, provided the frame for the manuscript, and critically revised the manuscript. SL and SM critically revised the manuscript. All authors read and approved the final manuscript.

## Conflict of Interest

The authors declare that the research was conducted in the absence of any commercial or financial relationships that could be construed as a potential conflict of interest.

## Publisher’s Note

All claims expressed in this article are solely those of the authors and do not necessarily represent those of their affiliated organizations, or those of the publisher, the editors and the reviewers. Any product that may be evaluated in this article, or claim that may be made by its manufacturer, is not guaranteed or endorsed by the publisher.
